# A comparative study of the structural-mechanical and porosity characteristics of multi-structure vascular scaffolds

**DOI:** 10.1038/s41598-025-34928-x

**Published:** 2026-01-06

**Authors:** Yao Lu, Jingbo Xu, Songbiao Xue, Xiaodi Zhou, Hongbo Zhang

**Affiliations:** 1 Zhejiang Pharmaceutical University,, Ningbo, 315100 Zhejiang China; 2 Ningbo Vercaty Medical Device Co., Ltd., Ningbo, 315100 Zhejiang China

**Keywords:** Vascular scaffold, Structural design, Manufacturing process, Mechanical properties, Structure-Property relationship, Pore geometry, Engineering, Materials science

## Abstract

The geometry of vascular scaffolds is a critical determinant of their clinical performance. However, the decoupled control over structural parameters (e.g., pore size, pore shape, and metal coverage) by different manufacturing processes (e.g., knitting, weaving, and stamping) and their systematic effects on mechanical properties remain unclear. This study aims to systematically compare multi-structured scaffolds fabricated by three distinct processes within a unified testing framework, revealing the intrinsic “process-structure-property” relationship. We designed and fabricated three series, totaling eight types, of tubular scaffolds from 316 L stainless steel: the S1 series (weft-knitted integrally formed), the S2 series (woven and rolled, with four mesh gradients: 30, 60, 90, and 120 mesh), and the S3 series (stamped and rolled, with three stamping-stretching ratios). Image analysis was employed to quantitatively characterize the pore area, pore shape (aspect ratio, circularity), and metal coverage of all scaffolds. Subsequently, their axial compression, axial tension, and radial support properties were systematically evaluated using an electronic universal testing machine. The results demonstrate that the different manufacturing processes successfully created a scaffold library with highly diverse structural parameters. he pore area spanned a wide range (from 0.22 mm² to 5.10 mm²). The S3 series (stamped structures) exhibited significantly anisotropic rhombic pores (aspect ratio 1.47–1.69), whereas the S1/S2 series featured near-isotropic elliptical/square pores (aspect ratio < 1.1). A core finding was the lack of a direct correlation between pore size and metal coverage across the series, confirming that structural parameters can be decoupled through different processes. Mechanical testing revealed that the S3 series exhibited the highest radial support force (peak force > 130 N), axial tensile strength (peak force up to 2447.75 N), and Apparent Compressive Stiffness (up to 135.36 N/mm). Conversely, the S1 (weft-knitted structure) showed the lowest stiffness and highest compliance (Apparent Compressive Stiffness 1.91 N/mm). The mechanical properties of the S2 (woven structures) were intermediate and varied systematically with increasing mesh count.

## Introduction

 The structural diversity of vascular scaffolds directly governs their clinical performance, including mechanical stability, hemodynamic behavior, and biological response^1,2^. Different geometric topologies, such as open-cell spirals, closed-cell sinusoidal waves, or mesh-like patterns, significantly influence key properties like flexibility, radial support force, bending stiffness, and vessel conformability. Comparative analysis of various structures under a unified testing framework is therefore essential to elucidate the interplay between scaffold geometry and the vascular environment, which can guide clinical selection for specific lesion contexts. Furthermore, modifications such as scaffold covering or drug-eluting coatings have proven effective in reducing restenosis and complications, highlighting the critical coupling between the underlying structure, surface properties, and drug release kinetics in determining final clinical outcomes^3,4^.

Existing literature on scaffold evaluation has predominantly focused on the mechanical or biological performance of single structural designs^5,6^.There is a notable gap in research that systematically compares mainstream structural classes under uniform conditions to reveal structure-property relationships. Moreover, the variability in materials, coatings, and experimental protocols across different studies makes direct comparison of their findings challenging^7,8^. This underscores the need for a comprehensive assessment of structural, porous, mechanical, and handling properties within a unified testing framework^9^. On the material front, factors such as the degradation control of magnesium alloys, the biocompatibility of polymer coatings, and their drug release kinetics demonstrate the coupled effect of structural design and material selection^10,11^. While these studies provide important insights, a systematic, cross-structural comparison remains largely unaddressed^12,13^.

Therefore, this study introduces a unified framework to compare a diverse library of scaffolds: a single baseline structure (S1), a family of structures with varying parameters (S2-1 to S2-4), and another distinct structural class (S3-1 to S3-3). We propose a “structure-porosity-mechanics” evaluation paradigm and test several verifiable hypotheses, such as the superior flexibility of mesh-like structures over open-cell designs, and the enhanced radial strength of closed-cell patterns. The objective is to enable quantitative cross-structural comparisons and identify performance trends, thereby providing a baseline and actionable predictive insights for optimal scaffold design and selection^14^.

The novelty of this work lies in the coupled analysis of pore architecture and surface characteristics, establishing systematic performance trends to guide scaffold selection and providing a critical baseline for subsequent research on coatings and drug delivery. The methodological foundation for our comparative framework is well-supported by literature; it has been shown that the mechanical behavior of braided scaffolds can be significantly tuned via design parameters, and the predictive accuracy of computational methods like finite element analysis (FEA) has matured considerably1^15,16^. Building on this, we will leverage existing data and graphical representations to offer a reproducible analytical pathway. The central novelty of this work lies in establishing a quantitative framework that demonstrates how distinct manufacturing processes can be leveraged to decouple traditionally linked structural parameters, such as pore size and metal coverage. Our final goal is to move beyond simple comparison and deliver practical design principles for optimizing the structure-material-surface combination tailored to diverse anatomical environments.

## Design and fabrication of metallic tubular scaffolds

### Design philosophy and theoretical basis

The structural design of a tissue engineering scaffold is a fundamental prerequisite that dictates its biological and mechanical performance. An ideal scaffold must not only provide requisite mechanical support but also feature a micro-architectural topology—defined by parameters such as pore size, shape, and porosity—that actively modulates cellular behavior and guides tissue regeneration. However, the precise tuning of scaffold performance is often constrained by the complex, coupled relationships among these critical structural parameters. For instance, a conventional design trade-off is that larger pore sizes are typically associated with higher porosity but lower mechanical strength, making it challenging to independently optimize these attributes.

To overcome this bottleneck, this study introduces a design strategy for decoupling structural parameters by leveraging distinct manufacturing processes. Instead of optimizing parameters within a single fabrication paradigm, we construct a multi-dimensional design space by employing three fundamentally different forming technologies: **weft knitting (Knitted)**, **weaving followed by rolling (Woven & Rolled)**, and **stamping followed by rolling (Stamped & Rolled)**. The core of this strategy is that each manufacturing process follows a unique physical logic in defining pore geometry and material distribution. This inherent difference allows us to “decouple” key parameters—such as pore size, shape, strut/wire width, and porosity—and achieve novel parameter combinations unattainable through any single conventional method.

Guided by this philosophy, we designed and fabricated three series of tubular metallic scaffolds, totaling eight distinct variants (designated S1, S2-1 to S2-4, and S3-1 to S3-3). This comprehensive set was developed to systematically investigate the deterministic influence of forming processes and their associated key structural parameters on the macroscopic performance of the scaffolds.

### S1 series scaffold: integrally Weft-Knitted

Design Objective: To fabricate a baseline scaffold characterized by high compliance, high toughness, and a three-dimensionally interconnected porous network.

#### Process selection

An integral weft-knitting technology was employed. This process forms a tubular structure through the continuous interlocking of metallic wire loops. Its non-fixed, looped junctions impart the scaffold with inherent flexibility and quasi-isotropic deformation capabilities.

#### Fabrication process

The S1 scaffold was fabricated using 316 L stainless steel wire with a diameter of 0.07 mm on a specialized small-bore knitting machine. By controlling knitting density and process parameters, a seamless tubular scaffold with a final diameter of 10 mm and a length of 15 cm was produced. This scaffold features a native, elliptically shaped pore architecture.

### S2 series scaffolds: woven mesh rolled and Welded, controlled by mesh count

#### Design objective

To construct a parametric gradient series with precisely predictable pore sizes and uniform square-shaped pores. This series is designed to isolate and investigate the influence of pore size as a single variable on scaffold performance.

#### Process selection

A weaving-then-rolling process was selected. The weaving technique allows for the precise and systematic control of pore size by modulating the density of warp and weft wires.

#### Fabrication process

First, plain-woven meshes of four different specifications—30, 60, 90, and 120 mesh—were produced on a loom using 316 stainless steel wire with a diameter of 0.06 mm. Subsequently, tailored sections of these meshes were precisely rolled onto a 10 mm diameter mandrel. The longitudinal butt seam was then secured using laser spot welding to form four distinct tubular scaffolds (designated S2-1, S2-2, S2-3, and S2-4), each with a uniform finished length of 15 cm. The defining characteristic of this series is its highly regular, square pore geometry, with pore area systematically decreasing as the mesh count increases.

### S3 series scaffolds: monolithic structure formed by Stamping and expansion

#### Design objective

To fabricate a series of scaffolds featuring a monolithic, continuous structure, high strength, and anisotropic pore characteristics.

#### Process selection

A stamping-then-rolling process was employed. This method begins with a solid metal sheet, which is stamped and expanded to form a continuous diamond-patterned mesh. This integral architecture eliminates the discrete wire junctions found in traditional woven or knitted structures, theoretically enhancing structural integrity and the efficiency of load transfer.

#### Fabrication process

Starting with a 0.50 mm thick 316 stainless steel sheet, three distinct expanded metal meshes with varying diamond-shaped apertures were created using three sets of precision dies, each with a different slitting-and-stretching ratio. These mesh sheets were then rolled into a tubular form and welded along the longitudinal seam. This process yielded three scaffold variants (S3-1, S3-2, and S3-3), each with a final diameter of 10 mm and a length of 10 cm. The hallmark of this series is its solid-state, continuous junctions, which form a robust, monolithic diamond-mesh network exhibiting pronounced mechanical anisotropy.

Through these three distinct design and fabrication pathways, we successfully established a scaffold library with a high degree of structural diversity. Table 1 summarizes the key design parameters for all eight scaffolds, laying a solid materials science foundation for subsequent performance characterization and structure-property relationship analysis.

**Table 1:** Design parameters and basic information of all scaffold groups.

Scaffold ID	Fabrication Process	Pore Geometry	Initial Wire/Sheet Diameter/Thickness (mm)	Structural Parameter (Mesh Count)	Final Dimensions (Diameter × Length)
S1	Integrally Knitted	Elliptical	0.07	N/A	10 mm × 15 cm
S2-1	Woven & Rolled	Square	0.06	30	10 mm × 15 cm
S2-2				60	10 mm × 15 cm
S2-3				90	10 mm × 15 cm
S2-4				120	10 mm × 15 cm
S3-1	Stamped & Expanded	Diamond	0.50	N/A	10 mm × 10 cm
S3-2				N/A	10 mm × 10 cm
S3-3				N/A	10 mm × 10 cm

### Specimen dimensions and normalization rationale

It is critical to note that the starting material (wire diameter or sheet thickness) and final scaffold dimensions (length, diameter) varied between the S1, S2, and S3 series, as detailed in Table 1. These variations are intrinsic to the different manufacturing processes and the available raw materials. For instance, the weft-knitting process (S1) utilized a 0.2 mm wire, while the woven meshes (S2) were sourced from stock with wire diameters ranging from 0.10 mm to 0.16 mm. This study does not aim for a direct, normalized comparison as if they were identical products, but rather to characterize and contrast the properties emerging from these distinct, real-world manufacturing routes. Our mechanical results should therefore be interpreted as properties of the entire structure, not as normalized material properties.

## Materials and methods

### Materials and equipment

#### Materials

The eight types of tubular metal scaffolds, categorized into three distinct series, were either custom-ordered or fabricated in-house for this study. Their key design parameters, including structural type, fabrication process, and material specifications, are detailed in Table 1.

#### Equipment

The following equipment was used for characterization and analysis:

##### Universal testing machine

Model UTM4304X (SUNS, Shenzhen, China).

##### Stereomicroscope

Model YYT-880E (Shanghai Yuanyi Optical Instrument Co., Ltd., Shanghai, China).

##### Image analysis software

ImageJ 1.53k (National Institutes of Health, NIH, USA).

### Experimental methods

#### Characterization of scaffold morphology and pore parameters

The macroscopic structure of all scaffolds was observed and photographed using a stereomicroscope (YYT-880E, Shanghai Yuanyi Optical Instrument Co., Ltd., Shanghai, China). To ensure representative sampling, at least three clear digital images were randomly captured from different locations for each scaffold type.

**Fig. 1:**
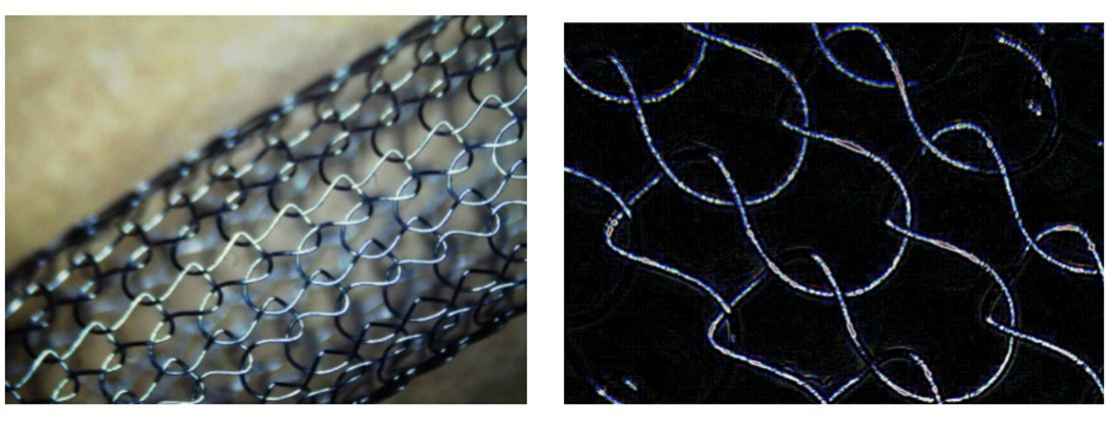
Representative image of a processed scaffold, using scaffold S1 as an example.

All captured images were subjected to quantitative analysis using ImageJ software (National Institutes of Health, USA). The analysis workflow was as follows: first, each image was calibrated according to its scale bar. Next, the pore regions were precisely distinguished from the metal struts using either manual or automatic thresholding. Finally, the built-in “Analyze Particles” tool was employed to measure each individual pore within the field of view, and the following morphological parameters were exported: Area (mm²), Major Axis (mm), Minor Axis (mm), Aspect Ratio (AR, calculated as Major Axis / Minor Axis), and Circularity (calculated as 4π × (Area / Perimeter²)). For each scaffold type, a minimum of 60 individual pores (over 20 pores per image) were measured for subsequent statistical analysis (Fig. 1).

#### Calculation of metallic coverage (MC)

Metallic Coverage (MC) was defined as the percentage of the area occupied by the metal struts in a two-dimensional projected image, serving as an indicator of scaffold density. This parameter was calculated from the stereomicroscope images using pixel statistics in ImageJ software. The formula is as follows:

1 $${\text{MC }}\left( \% \right){\text{ }}={\text{ }}({\text{Total Pixels}}\, - \,{\text{Pore Pixels}}){\text{ }}/{\text{ Total Pixels}} \times {\mathrm{1}}00\%$$

For each scaffold type, three images from different regions were analyzed to calculate the MC. The results were expressed as the mean ± standard deviation (Mean ± SD).

#### Mechanical property testing

All mechanical tests were performed on a Universal Testing Machine (Model UTM4304X, SUNS, Shenzhen, China).

(1) Radial Support Force Test.

To evaluate the mechanical support performance of the scaffolds in a simulated vascular environment, a radial compression test was conducted. Prior to testing, each scaffold sample was precisely positioned between two parallel compression plates. The machine applied radial compression at a constant rate of 1 mm/min until the scaffold’s diameter was compressed to 50% of its original value. Throughout the process, load-displacement data were recorded in real-time, and the deformation was documented using a camera. For each scaffold type, at least four parallel samples were tested (*n* ≥ 4). The peak force, representing the maximum radial force the scaffold could withstand before yielding or instability, was extracted from each load-displacement curve and used as the key indicator of its radial support strength.

(2) Axial Tensile Test.

To assess the mechanical response of the scaffolds under longitudinal loading, a uniaxial tensile test was performed. The two ends of each tubular scaffold sample were secured in custom fixtures to ensure that the tensile force was applied along the scaffold’s central axis. The test proceeded at a constant tensile rate of 10 mm/min until the sample fractured. The load-displacement data were recorded in real-time. For each scaffold specification, at least three parallel samples were tested (*n* ≥ 3). The Peak Tensile Force (N), defined as the maximum load the sample could sustain before fracture, served as the primary indicator of its axial tensile strength.

(3) Axial Compression Test.

Axial compression tests were conducted on 3 to 5 randomly selected parallel samples from each group (*n* = 3–5). The compression rate was set to 1 mm/min. The test was operated in a load-control mode, terminating when the applied load reached 2 N. Load-displacement data were automatically recorded by the system. From the axial compression test, an Apparent Compressive Stiffness (N/mm) was calculated. This value represents the structural stiffness of the entire scaffold rather than an intrinsic material modulus. It was determined by performing a linear regression on the initial, linear portion of the force-displacement curve, typically within the first 5–10% of axial strain, before the onset of significant buckling or non-linear deformation. This metric serves as a comparative measure of the scaffolds’ resistance to initial axial compression. The compressive displacement was defined as the maximum displacement value upon reaching the preset load (2 N) or structural failure. All results were presented as the mean ± standard deviation (Mean ± SD).

#### Statistical analysis and outlier handling

All quantitative data are presented as the mean ± standard deviation (Mean ± SD). During data processing, potential outliers were identified using the Grubbs’ test (α = 0.05). An outlier was defined as a data point resulting from an obvious experimental artifact, such as premature structural failure unrelated to the intended deformation mode.

In this study, one anomalous data point was identified in the S3-2 axial compression test, where a specimen exhibited an abnormally low modulus value inconsistent with its structural group. This data point was consequently excluded from the final analysis of the compressive modulus for the S3-2 group. No other data points were excluded or modified.

Statistical comparisons between groups were performed using a one-way analysis of variance (ANOVA) followed by Tukey’s HSD post-hoc test. A p-value of less than 0.05 (*P* < 0.05) was considered statistically significant. All statistical analyses and data plotting were performed using Origin 2024 software.4.

## Results and discussion

### Structural morphology and pore parameters of different scaffold series

**Fig. 2:**
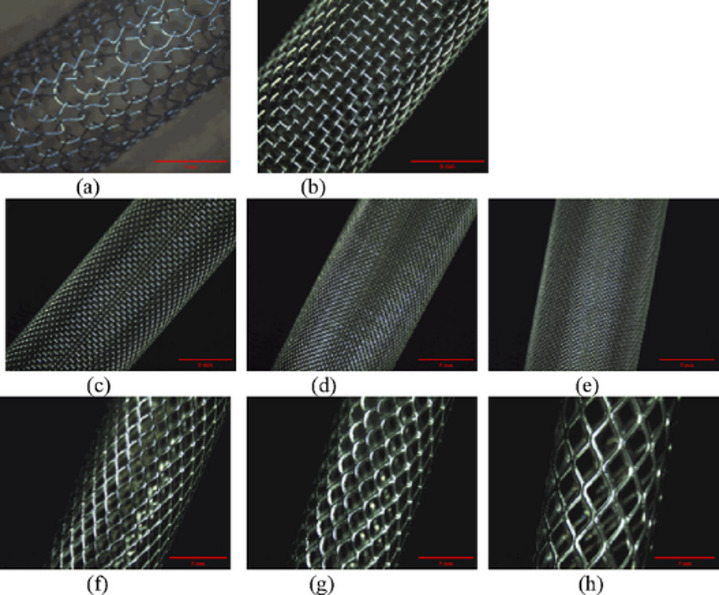
Macroscopic morphology of the eight metal scaffolds as observed by stereomicroscopy: (**a**) S1, (**b**) S2-1, (**c**) S2-2, (**d**) S2-3, (**e**) S2-4, (**f**) S3-1, (**g**) S3-2, and (**h**) S3-3.

Observations using a stereomicroscope revealed that the three scaffold series exhibited fundamental differences in their fabrication methods, resulting in distinctly different macroscopic structures (Fig. 2). The weft-knitted structure of S1 presented a flexible morphology composed of interconnected continuous loops. The woven structure of S2 displayed a regular, orthogonal mesh formed by the perpendicular intersection of warp and weft metal wires. In contrast, the stamped and expanded structure of S3 formed an integrated rhombic network, characterized by nodes of incompletely severed connections, which is typical of an expanded metal mesh. These distinct morphological differences provide the structural basis for the subsequent variations in their performance.

**Fig. 3:**
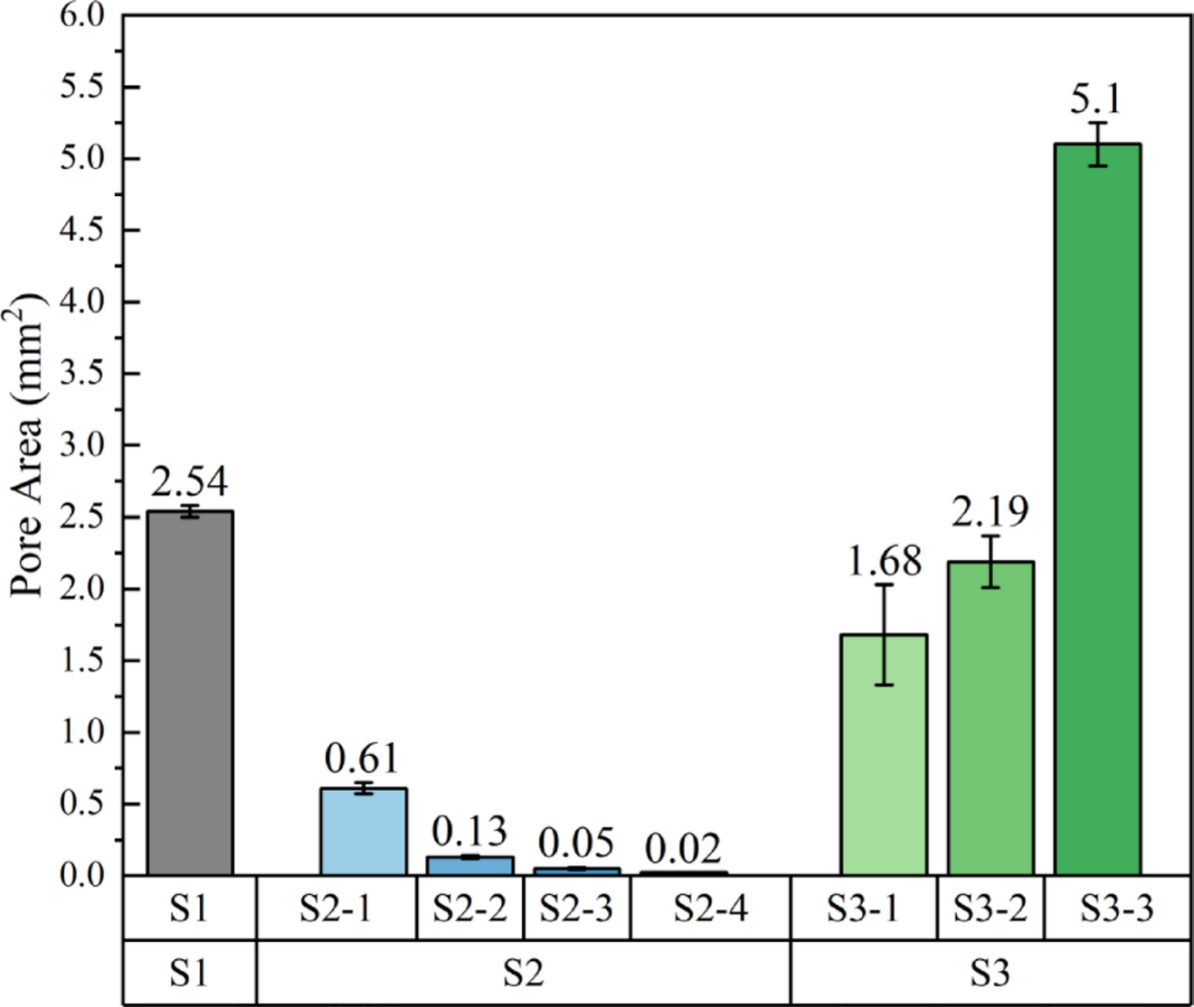
Pore geometry analysis of the eight scaffolds.

To quantify these morphological differences, a systematic analysis of the pore geometry was performed. As shown in Fig. 3, the results revealed that our study successfully constructed a scaffold library with pore areas spanning over two orders of magnitude, from a minimum of 0.22 ± 0.00 mm² (S2-4) to a maximum of 5.10 ± 0.35 mm² (S3-1). Such extensive coverage of pore sizes is essential for systematically examining the impact of this key physical parameter on biological responses. Clear pore size gradients were also established within each series.

S2 Series: The pore area showed a systematic decrease with an increasing mesh count (from 30 to 120 mesh), which is highly consistent with the theoretical model for woven meshes where pore size is inversely proportional to mesh count. This demonstrates that adjusting the mesh count is a precise and predictable method for controlling pore dimensions.

S3 Series: Similarly, controlling the stamping and expansion ratio enabled effective modulation of pore sizes, creating a gradient from small to large (S3-1 < S3-2 < S3-3). These well-defined intra-series gradients serve as ideal control sets for future studies.

**Fig. 4:**
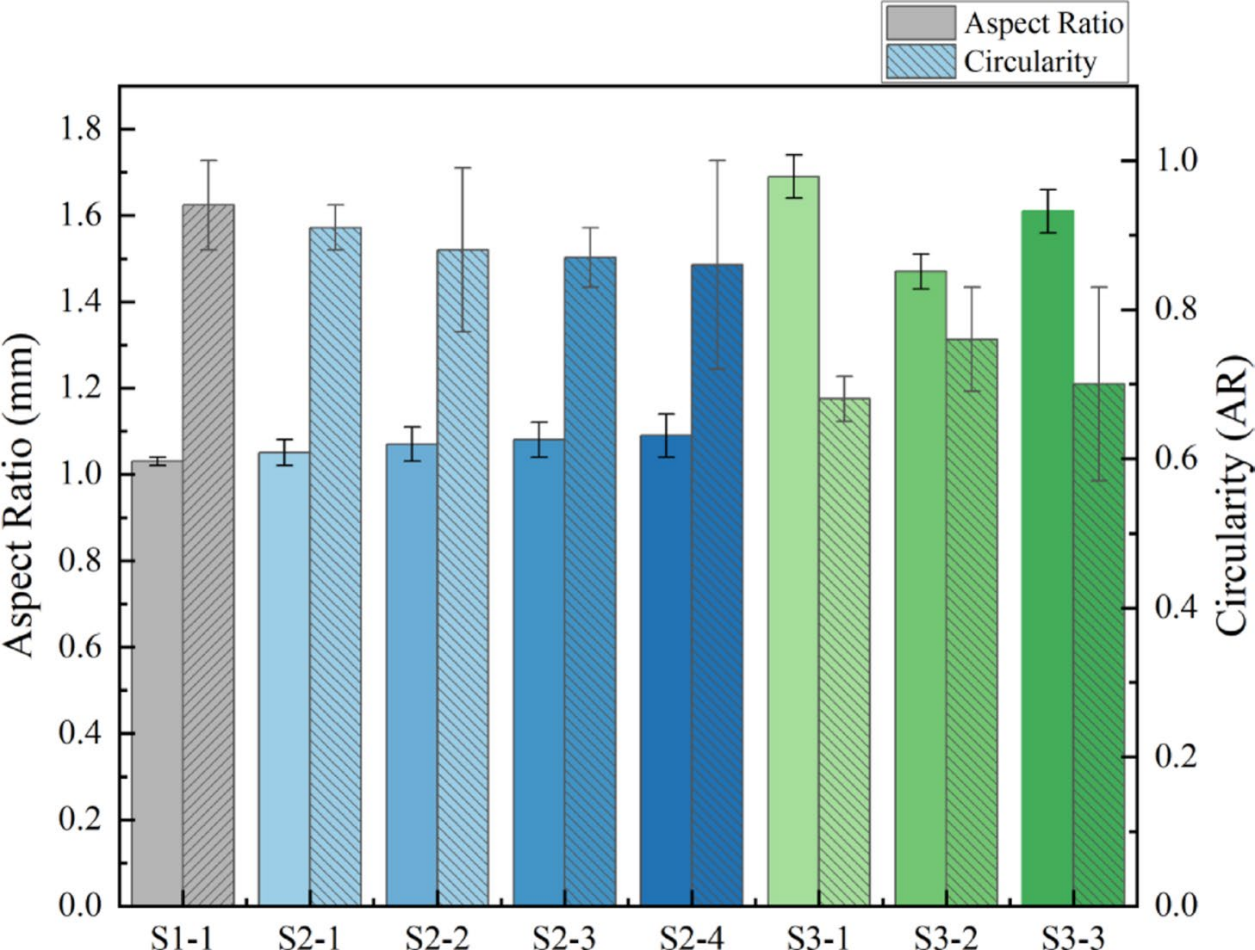
Comparison of pore shape parameters for the eight scaffolds.

Beyond pore size, pore geometry is a key structural parameter that profoundly affects cellular activities, including adhesion, migration, and differentiation. To quantify pore shape, we measured the aspect ratio (AR) and circularity, as shown in Fig. 4.

The S1 and S2 series both displayed AR values under 1.1 and circularity values typically exceeding 0.85. This suggests that their pores are largely isotropic (circular or square-like in 2D projection), which theoretically promotes uniform, non-directional tissue ingrowth. It is noteworthy that the AR in the S2 series slightly increased with the mesh count (from 1.05 to 1.09), which may be due to wire-to-wire interactions during dense weaving and rolling, causing a minor but detectable anisotropy.

In sharp contrast, the S3 series featured markedly elevated ARs (1.47–1.69) and reduced circularity (0.68–0.76), quantitatively defining the anisotropic nature of their diamond-shaped pores. These elongated, directionally oriented pores could offer topographical guidance, prompting cells to align and migrate along their major axis. This property is potentially valuable for applications where directional tissue regeneration is desired, such as for blood vessels, nerves, or ligaments.

### Control of metallic coverage and porosity via structural design

**Fig. 5:**
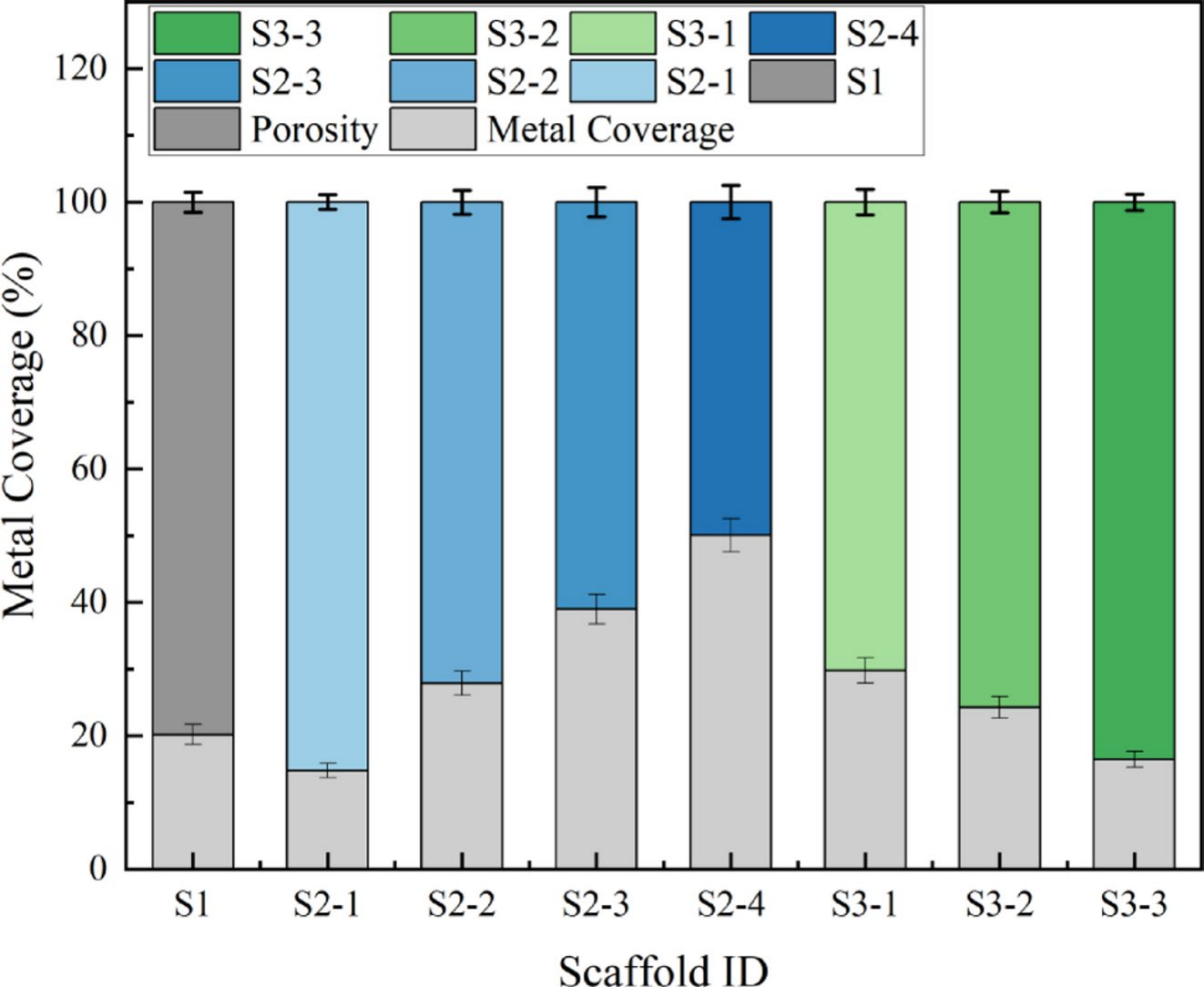
Metallic coverage and porosity for the eight types of scaffolds.

Metallic coverage (MC), a key indicator of scaffold openness, works in concert with porosity (defined as 100% - MC) to define the available space for cell adhesion and tissue ingrowth. These parameters also directly govern the efficiency of nutrient supply and waste removal. The relationship between MC and porosity for the eight scaffolds is clearly depicted in Fig. 5.

As anticipated, the metallic coverage within the S2 and S3 gradient series followed a predictable trend. When the wire diameter (0.06 mm for S2 series) or strut width (0.50 mm for S3 series) was held constant, decreasing the pore size necessarily resulted in a denser metallic framework per unit area. This systematically elevated the MC while reducing porosity. This effect was especially pronounced and consiscaffold in the S2 series, where porosity dropped sharply from a highly open 85.2% (S2-1) to a near semi-enclosed 49.9% (S2-4). This variation spans almost the full spectrum from high to low permeability, establishing a robust experimental foundation for identifying the optimal conditions for substance exchange and cellular invasion.

**Fig. 6:**
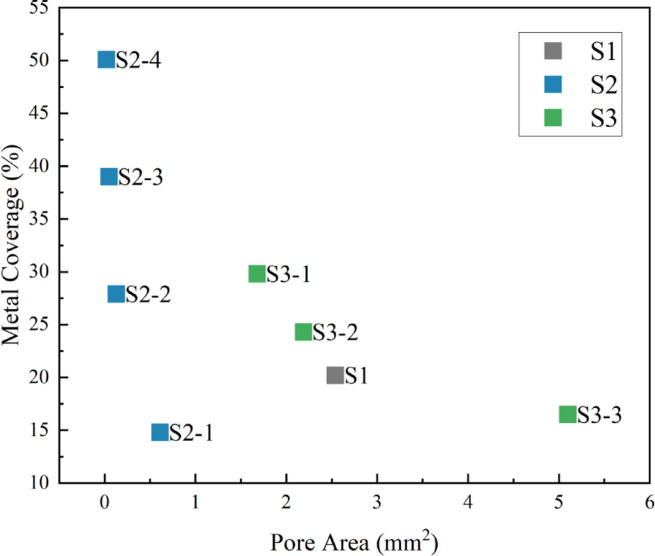
Correlation analysis of scaffold pore area and metallic coverage.

More importantly, when we correlate the key parameters of pore size and metallic coverage (Fig. 6), a crucial finding emerges: a simple inverse linear relationship between pore size and metallic coverage does not exist in cross-series comparisons. This observation contradicts the common intuition that larger pore size equates to higher porosity.As evident from the distribution in Fig. 6:

(1) S1 possesses a much larger pore area than S2-1 (2.54 mm² vs. 0.61 mm²), yet its metallic coverage is counterintuitively higher (20.2% vs. 14.8%). This discrepancy is due to the thicker wires (0.07 mm vs. 0.06 mm) and the loop-overlapping effect inherent in S1’s knitted architecture.

(2) While the pore area of S3-1 (1.68 mm²) far exceeds that of all S2 scaffolds, its metallic coverage (29.8%) is remarkably similar to that of S2-2 (27.9%), despite the latter having pores over ten times smaller. The primary reason is the substantial strut width of the S3 series (0.50 mm), which is much greater than the wire diameter of the S2 series (0.06 mm).

This non-linear relationship originates from the decoupled control of design parameters inherent in different manufacturing processes. The final porosity (or metallic coverage) of a scaffold is co-determined by “material parameters” (e.g., wire diameter, strut width) and “structural parameters” (e.g., mesh count, stretching ratio). The data distribution in Fig. 6 visually demonstrates that by combining different fabrication and structural parameters, we can achieve precise control over the scaffold’s microenvironment. For instance, it is possible to fabricate scaffolds with similar porosity but completely different pore sizes and shapes (e.g., S3-1 vs. S2-2). This provides an ideal and rigorous experimental model for subsequent functional studies to isolate and investigate the impact of a single structural variable—such as pore size, pore shape, or anisotropy—on biological outcomes. Consequently, this enables a more profound understanding of the structure-function relationship in tissue engineering.

### Characterization of mechanical properties

#### Radial support force

A primary function of the scaffold is to offer sufficient mechanical support for maintaining luminal patency. The radial support force of the various scaffold structures was quantified via radial compression testing, with the peak force statistics summarized.

in Fig. 7.

**Fig. 7:**
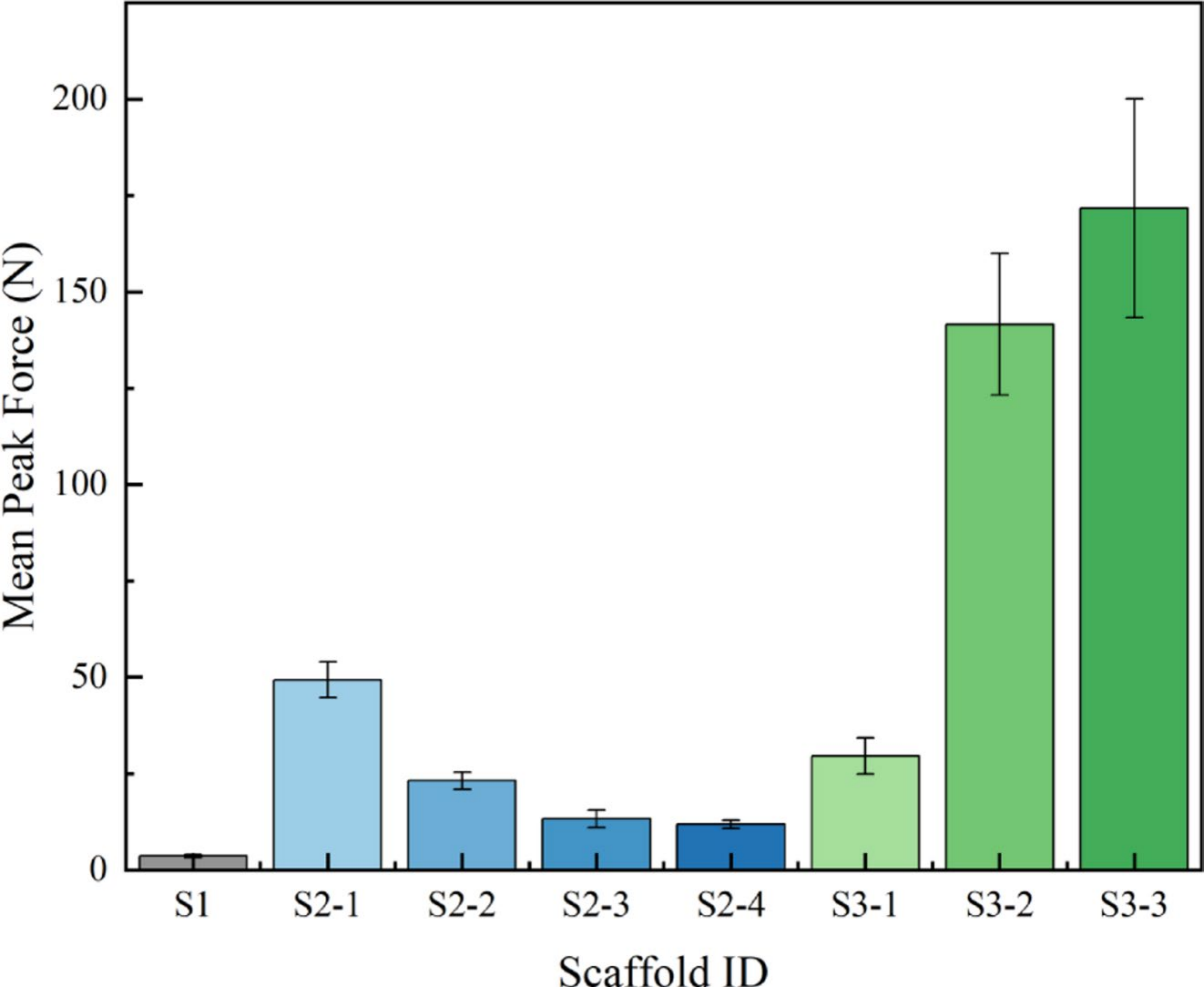
Comparison of radial support force (peak force) among the eight types of scaffolds.

As observed in Fig. 7, scaffolds with different fundamental structures exhibit vast differences in radial support force. The peak force ranges from approximately 3.6 N for S1 to over 170 N for S3-3, spanning a nearly 50-fold difference. This strongly indicates that structural design is the dominant factor determining the mechanical performance of the scaffolds.

Overall, the S3-structured scaffolds demonstrated the highest radial support force, with peak forces (137.56–171.68 N) far exceeding those of the S2 series (woven mesh, 12.98–49.32 N) and the S1 series (knitted mesh, 3.59 N). This significant disparity is primarily attributed to their structural integrity and material distribution:

S3 Series: Fabricated through a one-piece stamping and expansion process, its diamond-shaped mesh features continuous metallic connections at the nodes, forming a robust truss-like structure. Concurrently, its strut width (0.5 mm) is substantially larger than that of the other series, providing a large bending cross-section and thus enabling it to withstand immense radial pressure.

S2 Series: Composed of independent warp and weft wires interwoven together, with nodes secured by frictional forces. Under compression, slippage and rearrangement can likely occur between the wires, which may contribute to weaker structural rigidity.

S1 Series: The knitted loop structure is inherently flexible and prone to deformation, resulting in the lowest radial support force and exhibiting characteristics of high compliance.

Within the S2 and S3 series, the radial support force also follows clear patterns related to structural parameters, but the trends are diametrically opposed: S2 Series (Smaller pore size, weaker support force): From S2-1 to S2-4, as the mesh count increases and pore size decreases, the peak force systematically drops from 49.32 N to 12.98 N. This seemingly contradicts the intuition that denser structures should be stronger. The reason is that the overall rigidity of a woven mesh is primarily dictated by the bending resistance of individual wires and the long-range structural stability. While a smaller pore size—at a constant wire diameter (0.06 mm)—implies a shorter “unsupported length” for the wires, it also renders the overall structure more pliable and susceptible to buckling at a lower force.

S3 Series (Smaller pore size, stronger support force): From S3-1 to S3-3, as the pore size decreases, the peak force significantly increases from 137.56 N to 171.68 N. This aligns with expectations, as a smaller pore size—at a constant strut width (0.5 mm)—means more metallic struts and connection nodes per unit area. This makes the structure denser and markedly enhances its overall compressive stiffness.

#### Axial tensile properties and Structure-Performance relationship

In addition to resisting radial compression, scaffolds may also be subjected to complex loads such as tension and torsion during implantation and service. Therefore, axial tensile strength is another critical dimension of their mechanical reliability. In this study, the axial mechanical properties of the eight types of scaffolds were evaluated through uniaxial tensile tests.

**Fig. 8:**
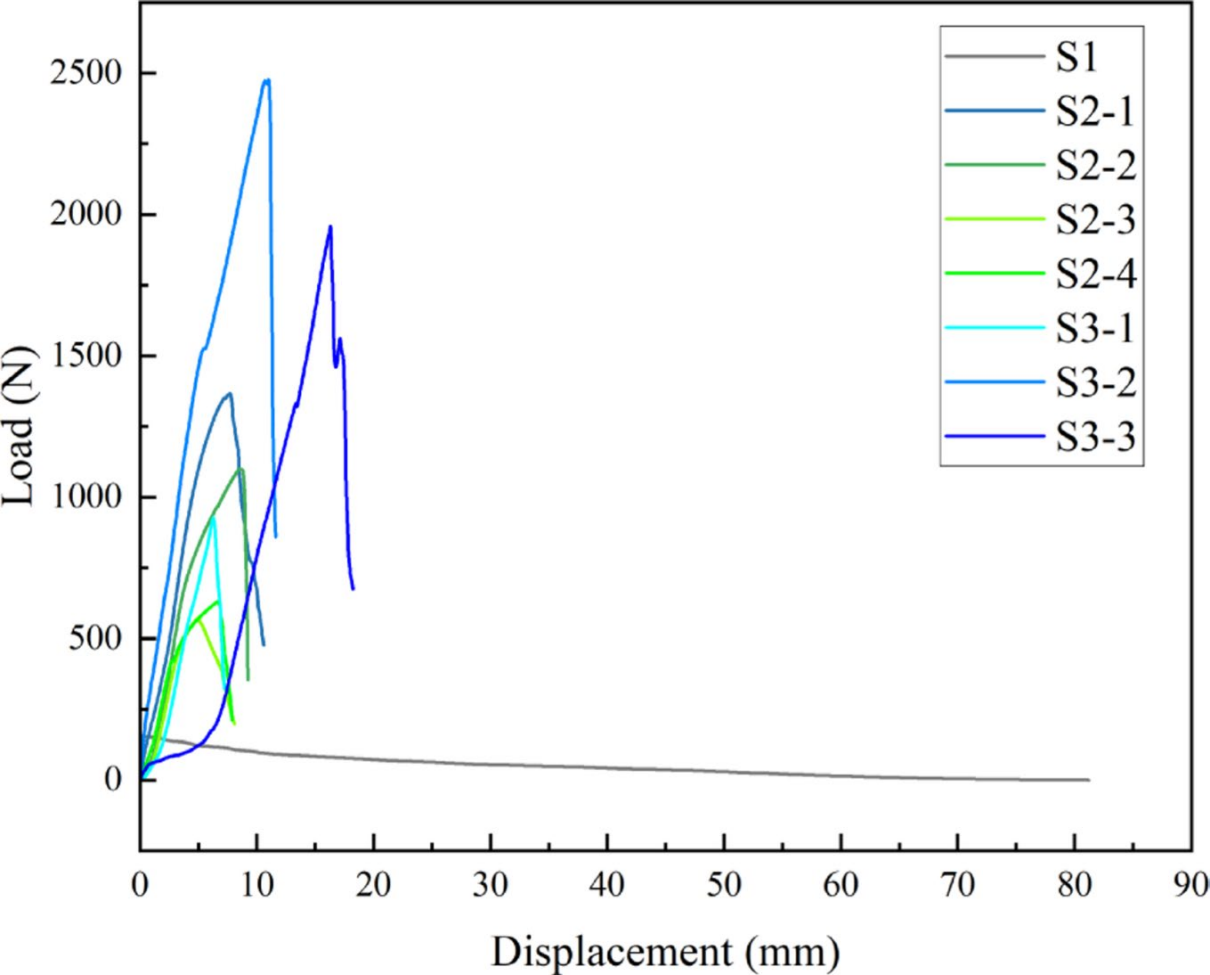
Representative axial tensile load-displacement curves for the eight scaffolds.

Figure 8 displays the representative tensile load-displacement curves for the eight scaffolds. All scaffolds exhibited a similar mechanical behavior pattern: an initial linear elastic stage where the load increased linearly with displacement, followed by a plastic deformation stage where the curve’s slope decreased. Finally, after reaching the peak load, the scaffolds underwent failure or fracture, leading to a rapid drop in load. However, significant differences were observed in the magnitude of the peak load, the corresponding displacement, and the overall shape of the curves among the different scaffolds. This reflects the decisive role of their internal structure in determining their mechanical behavior.

**Fig. 9:**
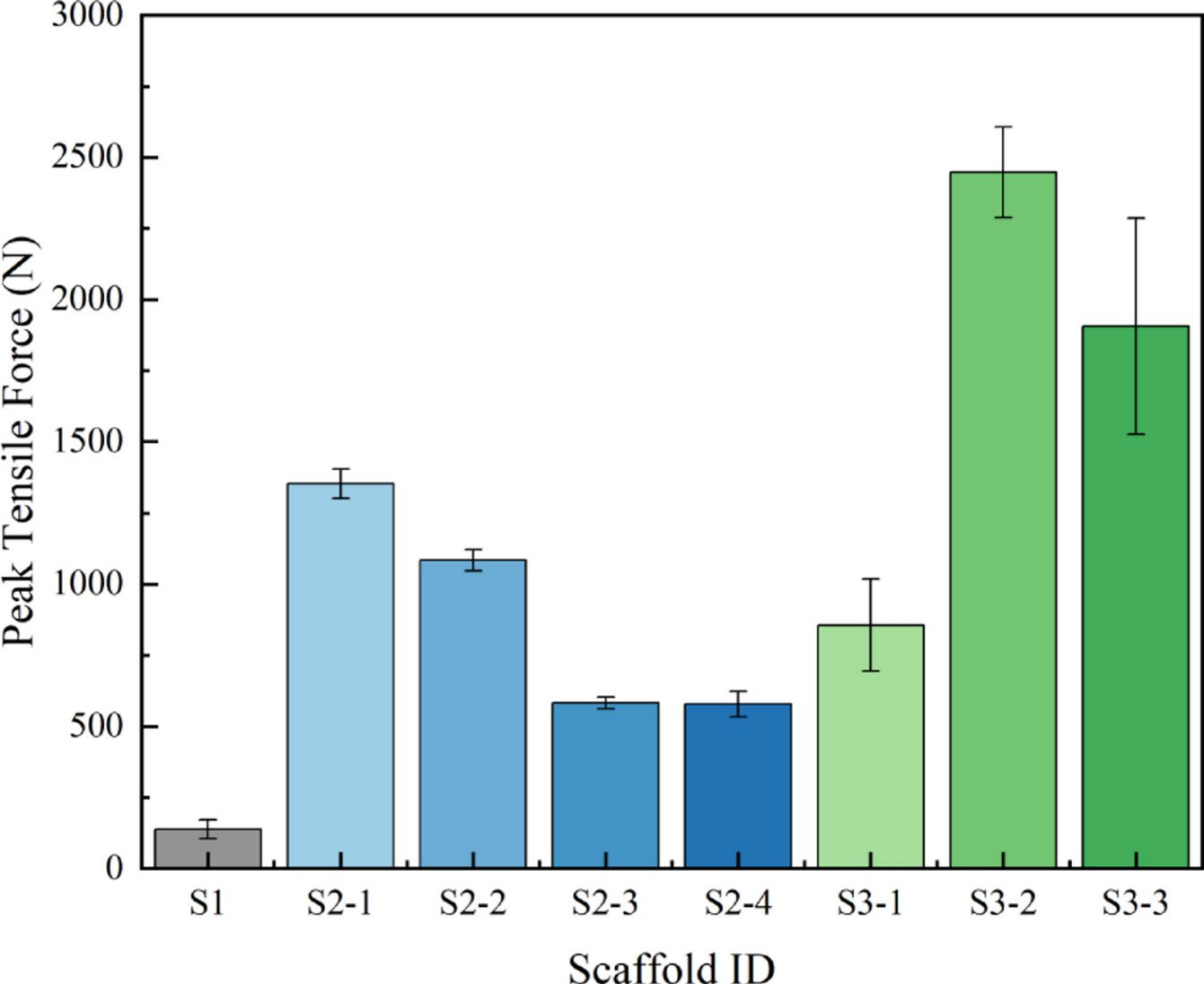
Comparison of axial tensile peak force among the eight types of scaffolds.

To quantitatively compare the axial tensile strength of each scaffold, we extracted their peak tensile forces, with the statistical results presented in Fig. 9. The data reveal a profound intrinsic relationship between scaffold structure and axial strength.

#### Manufacturing process has a decisive impact on axial tensile performance

Most notably, the S3 series (expanded metal mesh) demonstrated axial tensile strength far exceeding the other two series. Specifically, S3-2 reached an average peak force as high as 2447.75 N, nearly 18 times that of the S1 scaffold (138.48 N) and 1.8 times that of S2-1 (1352.26 N), the strongest in the S2 series. This vast difference stems from its monolithic structure formed by stamping and expansion. Compared to the discontinuous structures of S1 (interlocking loops) and S2 (interwoven warp and weft wires), the diamond-shaped cells of S3 are formed by continuous metal struts. During axial tension, the load can be effectively transferred and distributed through this continuous framework, enabling it to withstand greater pulling forces. This suggests that monolithic, continuous structures possess an inherent advantage in bearing axial tensile loads.

#### Strong correlation between axial Strength, pore Size, and metal coverage in the S2 woven mesh series

From S2-1 to S2-3, as the mesh count increased and pore size decreased, the peak tensile force systematically dropped from 1352.26 N to 583.03 N. This seems to contradict the intuition that “more material means more strength.” The underlying reason is that for a woven mesh, the axial tensile load is primarily borne by the longitudinal wires (warp). For a given total width, a higher mesh count leads to more individual warp wires. However, the resulting increase in total wire cross-sectional area (which is positively correlated with metal coverage) does not necessarily compensate for the strength reduction caused by an increased number of weave nodes and associated stress concentration points. An interesting turning point occurred between S2-3 and S2-4; although S2-4 has a higher mesh count and smaller pores, its tensile strength (578.58 N) was nearly identical to that of S2-3 (583.03 N). This may indicate that after reaching a certain critical weave density, the factors influencing axial strength become more complex, possibly reaching a plateau.

#### Non-linear strength variation within the S3 series

The S3 series also exhibited a non-linear change in strength. From S3-1 (856.1 N) to S3-2 (2447.75 N), the tensile strength increased sharply by nearly 2.9-fold. However, as the cell area further increased to S3-3 (5.10 mm²), the strength significantly decreased to 1905.44 N. This suggests that for an expanded metal mesh structure, there exists an optimal cell size-to-strut width ratio or geometric configuration (as exemplified by S3-2) that maximizes its axial load-bearing capacity. Pores that are either too small (S3-1) or too large (S3-3), this could potentially lead to uneven stress distribution or stress concentration at the nodes, which may in turn reduce the overall tensile strength. The structural parameter combination of S3-2 is clearly closer to this optimum point.

#### Comparison between axial tensile and radial support properties

By comparing the axial tensile performance (Fig. 9) with the previously discussed radial support performance (Fig. 7), we found no simple positive correlation between the two. For instance, S3-2 was the strongest in axial tension but weaker than S3-3 in radial support force. This profoundly reveals that the mechanical properties of the scaffolds are **anisotropic**—their mechanical responses in different directions are governed by different structural features. Radial support force is more dependent on the circumferential bending stiffness and structural stability, whereas axial tensile force relies more on the continuity and effective load-bearing cross-section of the longitudinal material. This mechanical anisotropy is a key consideration in functional scaffold design, allowing for customized structural optimization based on specific application scenarios (e.g., a vascular scaffold requiring high radial support vs. a ligament scaffold needing high axial strength).

#### Axial compression properties and Structure-Performance relationship of scaffolds

**Fig. 10:**
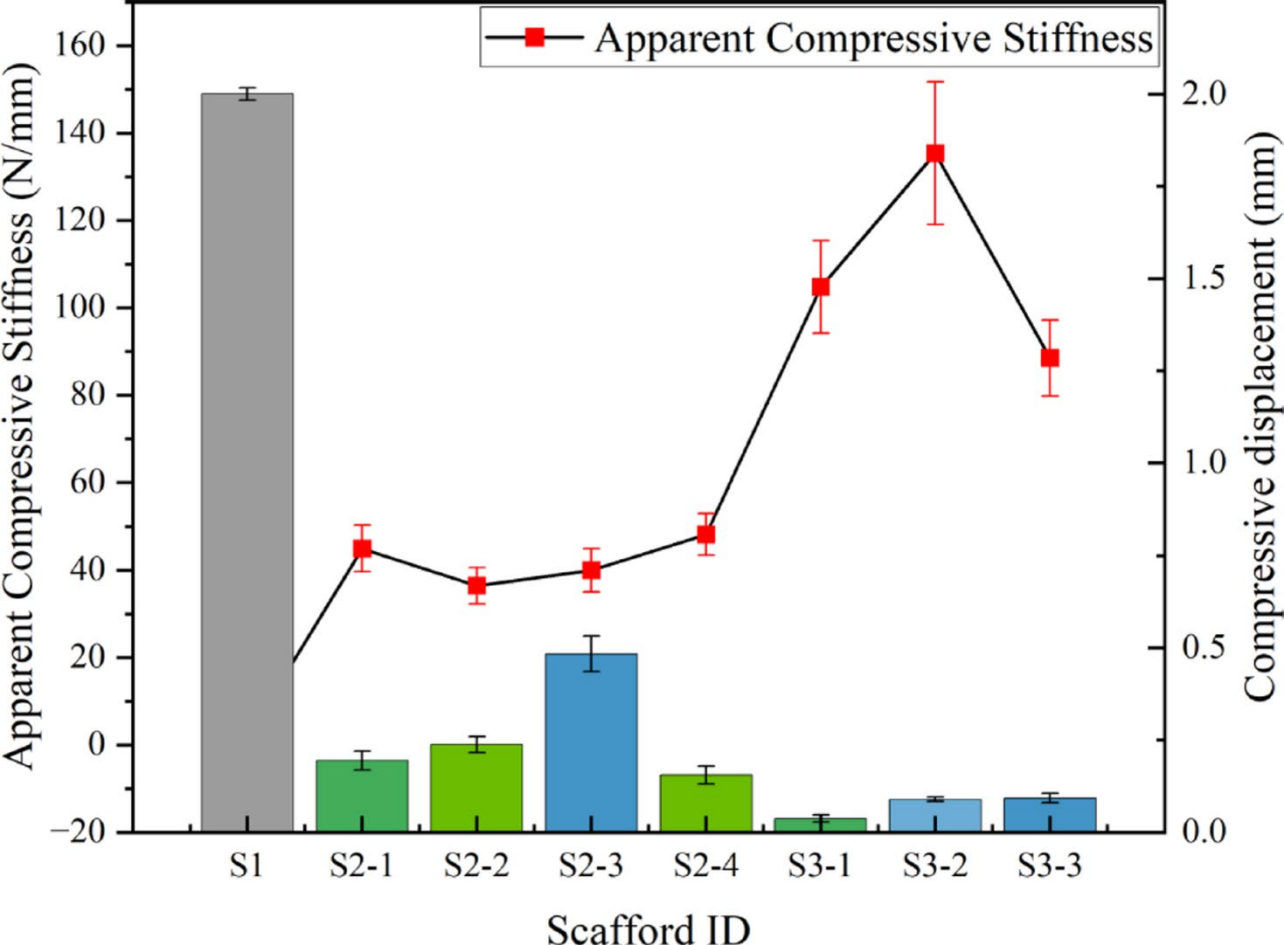
Comparison of axial compression properties among the scaffold groups. (**a**) Apparent Compressive Stiffness; (**b**) Compressive displacement.

**Fig. 11:**
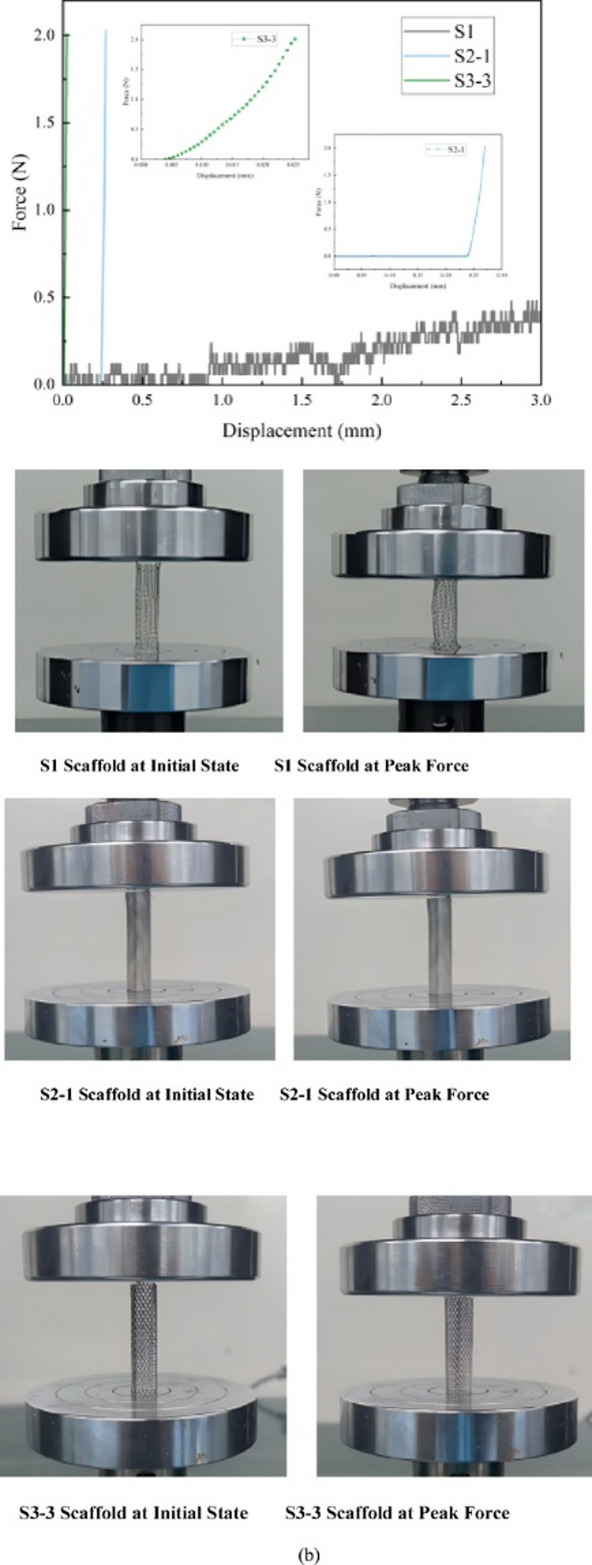
Representative load-displacement curves and deformation images of the scaffolds. (**a**) Typical load-displacement curves for S1, S2-1, and S3-3 scaffolds. (**b**) Corresponding deformation images at initial state, at peak force.

In this study, the axial compression properties of all eight groups of scaffolds—S1, the S2 series (S2-1 to S2-4), and the S3 series (S3-1 to S3-3)—were systematically evaluated. Figure 10 clearly illustrates the performance of each group in terms of two key mechanical indicators: Apparent Compressive Stiffness and compressive displacement. Furthermore, by analyzing the load-displacement curves and deformation videos (Fig. 11) recorded during the axial compression process, we can gain a deeper understanding of the failure modes of the different structures.

The test results in Fig. 10 clearly show that by altering the design parameters of the scaffolds, their mechanical properties exhibit a significant and graded variation. The Apparent Compressive Stiffness is a key indicator of a material’s ability to resist elastic deformation. As shown in Fig. 10(a), there are distinct and systematic differences in the Apparent Compressive Stiffness among the three types of scaffolds. The S1 scaffold, serving as a baseline structure, had the lowest modulus of only 1.91 ± 0.13 N/mm. This indicates high compliance and allows it to serve as a low-stiffness control. The four groups in the S2 series showed a substantial increase in stiffness, with their average moduli stabilizing within the range of 36–49 N/mm. Among them, S2-4 demonstrated the best performance in this series, with a modulus of 48.16 ± 4.78 N/mm.

Most prominently, the S3 series exhibited exceptional mechanical strength. The performance trend within this series became clear: the S3-2 scaffold achieved an astonishing Apparent Compressive Stiffness of 135.36 ± 16.35 N/mm, the highest value among all samples, followed closely by S3-1 (104.73 ± 10.57 N/mm). This result not only supports the notion that the superiority of the S3 series design but also reveals that the specific geometric configuration of S3-2 (such as an optimal cell size-to-strut width ratio) endows it with immense potential for axial load-bearing capacity, under both tensile and compressive conditions. This stepwise increase in modulus from S1 to S2 and then to S3 strongly demonstrates that our structural design strategy can precisely and effectively regulate the macroscopic mechanical strength of the scaffolds.

Compressive displacement, which reflects the scaffold’s deformation capability under load, showed a clear negative correlation with the Apparent Compressive Stiffness, as depicted in Fig. 10(b). The S1 scaffold could undergo a large deformation of up to 2.000 ± 0.017 mm under a specific load, consiscaffold with its low modulus characteristic. In stark contrast, the S2 and S3 series scaffolds, with their significantly enhanced stiffness, experienced a sharp decrease in compressive displacement. The S3 series, in particular, had the lowest magnitude of displacement, demonstrating a favorable combination of high stiffness and low deformation. It is noteworthy that S3-1 had the minimum compressive displacement (0.037 ± 0.009 mm) of all samples, indicating the strongest resistance to deformation, even though its modulus was slightly lower than that of S3-2.

## Study limitations

We acknowledge several limitations in this study that provide avenues for future research.

**First**, the sample size for mechanical testing (*n* = 3 to 5) was relatively small. This was a practical necessity due to the complex and resource-intensive custom fabrication of the eight distinct scaffold variants. While our results show clear and statistically significant trends between the major structural classes, a larger sample size would further increase the statistical confidence, particularly in quantifying the intra-series variability observed within the S3 group.

**Second**, our mechanical characterization was confined to quasi-static testing, including radial compression, axial tension, and axial compression. While these tests provide a fundamental understanding of the scaffolds’ primary mechanical properties, they do not capture the full dynamic loading environment experienced in vivo. Further investigations involving fatigue, bending, and cyclic loading are necessary to fully assess the long-term durability and predict the long-term performance of these structures.

**Third**, this study did not include a computational approach, such as Finite Element Analysis (FEA), to analyze local stress and strain concentrations. Such an analysis would be a valuable complementary step to provide deeper mechanistic insights into the observed failure modes and to optimize strut-level geometry more efficiently.

**Finally**, the scope of this work was focused on the “process-structure-property” relationship from a mechanical and structural perspective. The biological response—including hemocompatibility, endothelialization, and tissue ingrowth—is critical for clinical success and warrants comprehensive investigation in subsequent studies built upon the foundational data presented here.

Furthermore, the direct comparison between manufacturing series is inherently confounded by variations in starting material dimensions (e.g., wire vs. sheet thickness) and final scaffold geometry. Our study characterizes the properties of these distinct structural systems as-is, rather than attempting to normalize for all geometric variables, which would be impractical. The findings highlight the distinct characteristics of each process-derived structure.

## Conclusions

Through a systematic structure-performance comparison of eight metal scaffolds fabricated by three manufacturing processes (weft-knitting, weaving, and stamping-expansion), the following conclusions were drawn:

**The manufacturing process determines the fundamental structural features and the upper limit of a scaffold’s performance.** The stamping-expansion process (S3 series) creates an integrated diamond-mesh network structure that exhibits significant advantages in mechanical properties in radial support force, axial tensile strength, and Apparent Compressive Stiffness. This makes it highly suitable for clinical scenarios requiring robust mechanical support.

**The weft-knitting process (S1 series) imparts inherent compliance to the scaffolds.** Their non-fixed, interlocking loop structure results in the lowest mechanical modulus and the highest deformability, which could be potentially valuable in applications requiring excellent conformability with tortuous blood vessels.

**The weaving process (S2 series) offers an intermediate platform with tunable properties.** By precisely controlling the mesh count, it is possible to systematically regulate the scaffold’s cell size, metal coverage, and corresponding mechanical properties, thus providing a flexible solution for customized scaffold design.

**A complex**,** non-linear relationship exists between the structural parameters and mechanical properties of the scaffolds.** Notably, a smaller cell size does not always translate to higher strength. Performance anisotropy (i.e., the difference between radial and axial properties) is also significant, highlighting the importance of multi-dimensional structural optimization tailored to specific application scenarios.

In summary, this study not only quantifies the performance differences among various scaffold structures but, more importantly, reveals the central role of the manufacturing process in decoupling and controlling key structural parameters. It provides a solid experimental foundation and design principles for the future rational design of functionalized and personalized vascular scaffolds.

Beyond summarizing performance differences, this study provides a practical design roadmap. For applications demanding high radial support, a stamped and expanded structure (like the S3 series) offers a superior starting point. Conversely, for anatomical sites requiring high flexibility and conformability, such as tortuous vessels, a weft-knitted design (S1) is more appropriate. Most importantly, our cross-series analysis demonstrates that achieving a specific porosity does not predetermine pore size or mechanical strength; these can be tuned independently by selecting the appropriate manufacturing process. This insight allows designers to break free from traditional trade-offs, enabling the creation of scaffolds with novel combinations of properties tailored for specific clinical needs.

## Data Availability

All data generated or analysed during this study are included in this published article. The raw data underlying the final reported results are available from the corresponding author upon reasonable request.
